# Pregnancy-Related Acute Kidney Injury in Spontaneous Ovarian Hyperstimulation Syndrome: A Case Report

**DOI:** 10.7759/cureus.96132

**Published:** 2025-11-05

**Authors:** Yeshwanth Mohan Yelavarthy, Somnath Panda, Jyoti Aggarwal, Subramanian Narayanan Kaladi, Vinay Rathore

**Affiliations:** 1 Nephrology, All India Institute of Medical Sciences, Raipur, Raipur, IND; 2 Internal Medicine, All India Institute of Medical Sciences, Raipur, Raipur, IND

**Keywords:** acute kidney injury, dialysis, pregnancy loss, spontaneous ovarian hyperstimulation syndrome, third spacing

## Abstract

Spontaneous ovarian hyperstimulation syndrome (OHSS), a rare entity distinct from iatrogenic forms, presents a diagnostic challenge, particularly in early pregnancy, often leading to delayed recognition and severe systemic complications. We present this case to highlight an unusual and life-threatening presentation of spontaneous OHSS complicated by severe acute kidney injury (AKI) requiring dialysis in early pregnancy, a scenario seldom reported in the literature. A 27-year-old Gravida 2 Para 1 Living 1 (G2P1L1) woman at 10 weeks of spontaneous gestation, with no history of assisted reproductive technology, presented with generalised oedema, reduced urine output, and vaginal bleeding, progressing to multi-organ fluid sequestration, pregnancy loss (missed abortion), and AKI requiring dialysis. Management included aggressive fluid resuscitation, three sessions of hemodialysis for uraemia and severe metabolic acidosis, and therapeutic paracentesis/thoracentesis. Clinicians should maintain a high index of suspicion for OHSS in early gestation with unexplained third spacing and renal dysfunction, and timely fluid management, including renal replacement therapy when indicated.

## Introduction

Ovarian hyperstimulation syndrome (OHSS) is a potentially life-threatening complication following Assisted Reproductive Technology (ART) and in vitro fertilisation (IVF). It characteristically manifests as cystic ovarian enlargement and massive fluid shifts into the third space due to increased vascular permeability [1]. Severe OHSS occurs in less than 2% of ovarian stimulation cycles. Most contemporary studies report a prevalence of 0.1-2% [2,3]. Occasionally, even without a known trigger of ART, OHSS can occur, and this is termed spontaneous OHSS. It is a diagnostic challenge in the absence of a typical ART history, often leading to a delay in recognition. It often presents within the first eight to 14 weeks of pregnancy and manifests with systemic features such as ascites, pleural/pericardial effusions, oliguria, and electrolyte disturbances. If unrecognised, it usually rapidly progresses to fulminant multi-organ dysfunction [1,2]. This case showcases a rare, life-threatening presentation of severe spontaneous OHSS in early pregnancy, complicated by multiorgan fluid sequestration, acute kidney injury (AKI), and pregnancy loss.

## Case presentation

**Figure 1:**
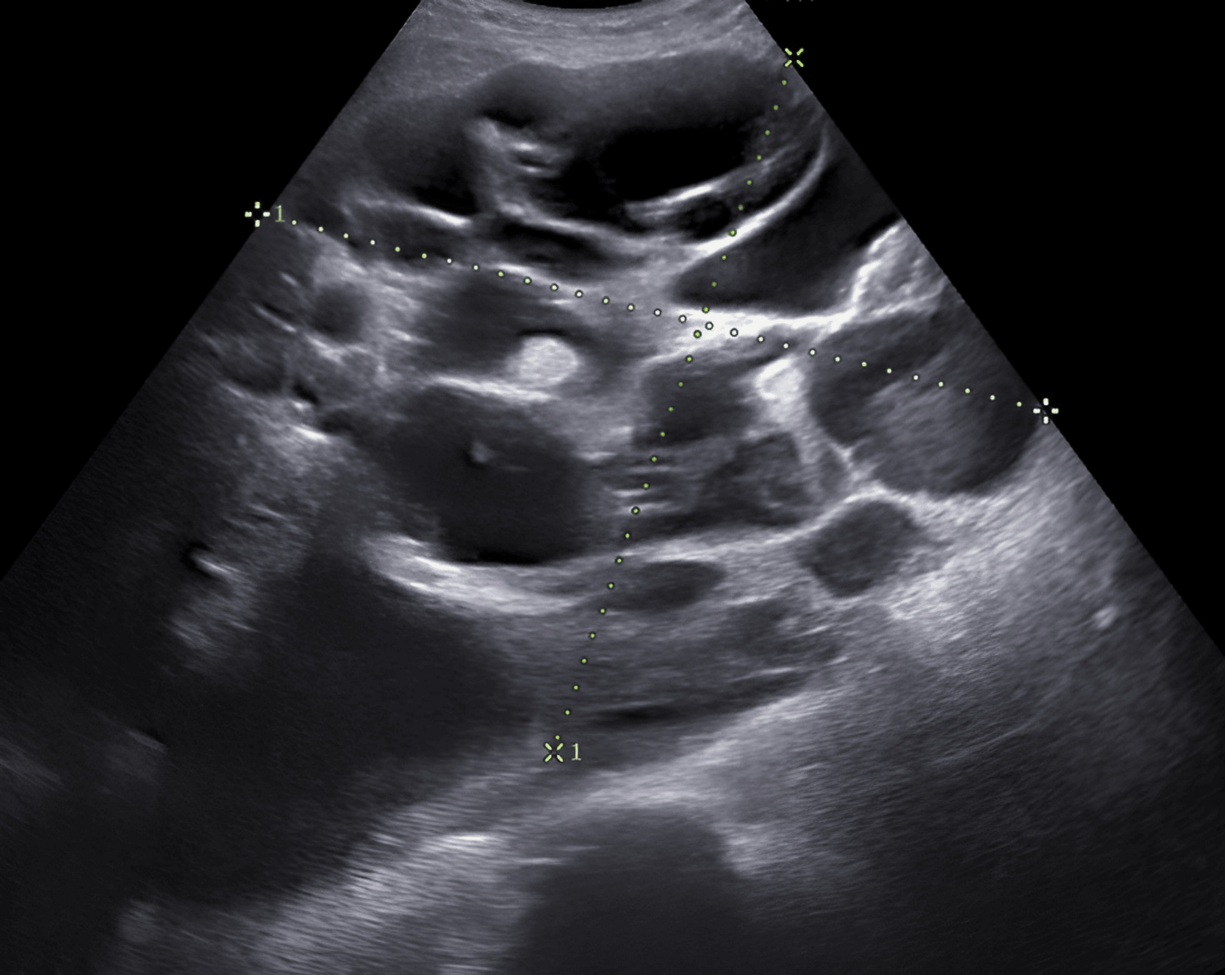
Transabdominal pelvic ultrasound It shows a markedly enlarged right ovary measuring 13.74 × 12.43 cm, with multiple large thin-walled cysts.

A 27-year-old Indian woman, Gravida 2 Para 1 Living 1 (G2P1L1), at 10 weeks of gestation, presented with complaints of generalised body swelling, reduced urine output for seven days, and vaginal bleeding for the past three days. She had conceived spontaneously and had no history of ART in her previous pregnancy. At admission, she had tachycardia (pulse rate: 124 bpm; reference: 60-100 bpm), tachypnea (respiratory rate: 32 breaths per minute; reference: 12-20 breaths per minute), and a blood pressure of 85/44 mmHg (reference: systolic blood pressure of <120-90 mmHg; diastolic blood pressure of <90-60 mmHg). Mild pallor and generalised oedema were noted. Abdominal examination revealed uniform distension with a positive fluid thrill. On bimanual examination, the uterus was enlarged, consistent with a gestational age of 10 weeks. Local genital examination revealed spotting of dark red blood with a closed cervical os. Ultrasound abdomen evaluation revealed massive ascites and an enlarged uterus with an empty gestational sac and no fetal pole or cardiac activity. Both ovaries were grossly enlarged (right ovary: 134 × 124 × 92 mm; left ovary: 128 × 112 × 85 mm), with multiple thin-walled cysts and septations, with no internal echoes, as shown in Figure 1.

Lung ultrasound revealed bilateral pleural effusion. Transthoracic echocardiography revealed mild pericardial effusion. Initial laboratory investigations (Tables 1, 2) revealed grossly deranged serum creatinine (13.46 mg/dL) and urea (272 mg/dL).

**Table 1:** Laboratory investigations of the patient at presentation BUN: blood urea nitrogen; PT: prothrombin time; INR: international normalised ratio; aPTT: activated partial thromboplastin time; TSH: thyroid-stimulating hormone; HbA1c: glycated haemoglobin; AFP: alpha-fetoprotein, CA-125: cancer antigen-125; CEA: carcinoembryonic antigen; β-hCG: beta-human chorionic gonadotropin; ANA: antinuclear antibody; APLA: antiphospholipid antibody; PR3: proteinase 3; MPO: myeloperoxidase.

Parameter	Patient’s value	Reference range	Interpretation
Serum electrolytes			
Sodium (Na⁺)	124 mEq/L	135-145 mEq/L	Hyponatraemia
Potassium (K⁺)	5.3 mEq/L	3.5-5.0 mEq/L	Mild hyperkalaemia
Chloride (Cl⁻)	<80 mEq/L	98-106 mEq/L	Marked hypochloraemia
Ionised Calcium (iCa²⁺)	1.05 mmol/L	1.12-1.32 mmol/L	Mild hypocalcaemia
Renal function			
Creatinine	13.46 mg/dL	0.6-1.2 mg/dL	Severe renal impairment
Urea (BUN)	272 mg/dL	7-20 mg/dL	Severely elevated (Uraemia)
Liver function			
Total protein	5.2 g/dL	6.0-8.3 g/dL	Low (Hypoproteinaemia)
Albumin	1.8 g/dL	3.5-5.0 g/dL	Marked hypoalbuminaemia
CBC (Complete Blood Count)			
Haemoglobin	9.8 g/dL	12-16 g/dL (F), 13-17 g/dL (M)	Anaemia
Haematocrit	30%	36-46% (F), 41-53% (M)	Anaemia
Total leukocyte count (TLC)	11,730 /µL	4,000-11,000 /µL	Mild leukocytosis
Platelets	210 × 10³/µL	150-450 × 10³/µL	Normal
PT	13 sec	11-15 sec	Normal
INR	1.0	0.8-1.2	Normal
aPTT	31 sec	25-35 sec	Normal
Endocrine & tumor markers			
TSH	3.8 μIU/mL	0.4-4.5 μIU/mL	Normal
HbA1c	5.4%	<5.7%	Normal (non-diabetic)
AFP	0.3 ng/mL	<10 ng/mL	Normal
CA-125	169.8 U/mL	<35 U/mL	Significantly elevated
CEA	0.95 ng/mL	<3 ng/mL (non-smoker)	Normal
β-hCG	<2 mIU/mL	<5 mIU/mL	Normal
Autoimmune markers			
ANA	Negative	Negative	Normal
APLA workup	Negative	Negative	Normal
PR3 & MPO (ELISA)	Negative	Negative	Normal

**Table 2:** Analyses of the patient's ascitic and pleural fluids ADA: adenosine deaminase; LDH: lactate dehydrogenase; TB: Tuberculosis; AFB: acid-fast bacilli; CBNAAT: cartridge-based nucleic acid amplification test.

Parameter	Patient’s value	Reference range / expected	Interpretation
Ascitic fluid analysis			
Appearance	Clear, straw-colored	Normally clear, pale yellow	Normal
Total protein	1.6 g/dL	<2.5 g/dL (transudate), >2.5 g/dL (exudate)	Low → Transudative ascites
SAAG (Serum–Ascites Albumin Gradient)	0.7 g/dL	≥1.1 g/dL (portal hypertension), <1.1 g/dL (non-portal hypertension)	Low (<1.1) → non-portal hypertensive cause
Cell count	180 cells/mm³ (N20%, L80%)	<500 WBC /µL	Lymphocyte-predominant, no spontaneous bacterial peritonitis
ADA	20 U/L	<30–40 U/L (not suggestive of TB)	Normal, not consistent with TB
LDH	180 U/L	<200 U/L (normal range, varies)	Normal
Glucose	85 mg/dL	Similar to serum level (>50 mg/dL)	Normal
Cytology	Negative	Negative	Normal
Gram stain/Culture	No organisms, sterile	Sterile in non-infective cases	Normal
AFB	Negative	Negative	Normal
GeneXpert/CBNAAT	Negative	Negative	Normal
Pleural fluid analysis			
Appearance	Clear, pale yellow	Normally clear, pale yellow	Normal
Total Protein	1.5 g/dL	<3.0 g/dL (transudate), >3.0 g/dL (exudate)	Low → Transudative pleural effusion
Cell Count	200 cells/mm³ (lymphocyte-predominant)	<1000 cells/mm³ (non-purulent); neutrophils if acute infection, lymphocytes if chronic/TB/malignancy	Mild elevation, lymphocyte-predominant
ADA	22 U/L	>40 U/L suggests TB	Normal, not TB
LDH	190 U/L	<200 U/L (transudate), >200 U/L (exudate by Light’s criteria)	Borderline, favors transudate
Glucose	88 mg/dL	Similar to serum (>60 mg/dL)	Normal
Cytology	Negative	Negative	Normal
Gram stain/Culture	No organisms, sterile	Sterile in non-infective cases	Normal
AFB	Negative	Negative	Normal
GeneXpert/CBNAAT	Negative	Negative	Normal

Blood gases revealed severe metabolic acidosis (pH 7.10, HCO₃⁻ 12 mEq/L, anion gap 18 mEq/L) with hyperkalaemia (K^+^ 5.5). There was also profound hypoalbuminaemia (1.8 g/dL).

Given the clinical picture of anasarca, third spacing with hypoalbuminaemia, and enlarged multicystic ovaries in early pregnancy, a provisional diagnosis of OHSS, complicated by pregnancy loss and AKI was considered. She underwent initial fluid resuscitation with isotonic crystalloids followed by colloids (human albumin) with diuretics. Subsequently, she underwent three sessions of hemodialysis in view of uraemia and severe metabolic acidosis. Therapeutic paracentesis and thoracentesis were performed, yielding symptomatic relief. She was discharged after two weeks at a stable creatinine of 1.8 mg/dL. At the one-month follow-up, her serum creatinine had improved to 0.9 mg/dL.

## Discussion

OHSS is an iatrogenic complication most frequently associated with exogenous gonadotropin administration for ovulation induction in ART. However, spontaneous OHSS, occurring without such intervention, is rarely reported and often linked to elevated endogenous human chorionic gonadotropin (hCG) levels, as seen in molar pregnancies or multifetal gestations, hypothyroidism, or mutations in the FSH receptor (FSHR) gene [4,5]. This case did not have these risk factors. FSHR hyperresponsiveness, which amplifies the ovarian response to physiologic hCG levels in early pregnancy, can precipitate OHSS without exogenous stimulation and may contribute to its persistence even after hCG levels fall, as in our case. This leads to increased vascular permeability and third-space fluid accumulation, primarily in the peritoneal and pleural cavities, leading to hemoconcentration, electrolyte imbalances, and reduced organ perfusion [6]. AKI could be a consequence of severe hypovolaemia, direct renal parenchymal injury from pro-inflammatory cytokines, or microthrombosis within the renal vasculature due to haemoconcentration and hypercoagulability [2]. Clinical features include mild abdominal distension, nausea, vomiting, and breathlessness, which often resolve within two weeks, or they can rapidly progress to life-threatening complications such as massive ascites, pleural or pericardial effusions, and AKI, as in our case [4]. 

Diagnosis relies on clinical assessment, supported by targeted imaging and laboratory evaluation. Pelvic and abdominal ultrasound is the cornerstone, revealing bilaterally enlarged cystic ovaries and ascites. Additionally, in suspected severe OHSS, urinalysis and urine microscopy have been useful for assessment of proteinuria and to exclude infection or glomerular pathology as possible etiologies of AKI [2]. However, this was not deemed to be useful in our case, as she was oliguric. Serum studies, including haematocrit and osmolality, are essential to identify haemoconcentration and third spacing. In this case, elevated cancer antigen (CA)-125 and large cystic ovaries (>10 cm) raised initial concern for malignancy; however, classic imaging features of OHSS (thin-walled, septate cysts) and transudative ascites helped avoid unnecessary laparotomy [7]. 

Management of AKI, in the context of severe or critical OHSS, centers on supportive care aimed at restoring and maintaining intravascular volume [5]. Isotonic crystalloids are mainstays for initial resuscitation, improving renal perfusion and potentially neutralising vascular permeability factors. Despite aggressive fluid resuscitation, our patient remained oliguric. We hypothesised intrinsic renal injury, although a renal biopsy was not performed. Data regarding abortion in pregnancies complicated by spontaneous OHSS are limited. Previous literature has shown higher rates of abortion in IVF-associated OHSS; however, our case had an adverse event of missed abortion despite it being a pregnancy as a result of spontaneous conception [8].

## Conclusions

Clinicians should consider the possibility of spontaneous OHSS even in the absence of fertility treatment, particularly in early pregnancy patients presenting with AKI, abdominal distension, nausea, or vomiting. Ultrasonography plays a pivotal role in establishing the diagnosis by demonstrating bilaterally enlarged, cystic ovaries and excluding other differential causes, thereby helping to avoid unnecessary interventions. Once diagnosed, early and aggressive volume resuscitation remains the cornerstone of management. In severe cases, dialysis serves as a temporary supportive measure to correct uraemia, electrolyte disturbances, and acid-base imbalances until the underlying pathophysiological process resolves spontaneously.
